# Supplementary material for: Tissue-specific transcriptome responses to Fusarium head blight and Fusarium root rot
Source: Front Plant Sci. 2022 Oct 24;13:1025161. doi: 10.3389/fpls.2022.1025161 (PMC9637937; doi:10.3389/fpls.2022.1025161)
Supplement: Supplementary file 3 [file DataSheet_3.docx]

This figure supplements the following manuscript:

Tissue-specific transcriptome responses to Fusarium head blight and Fusarium root rot

By John F. Haidoulis and Paul Nicholson


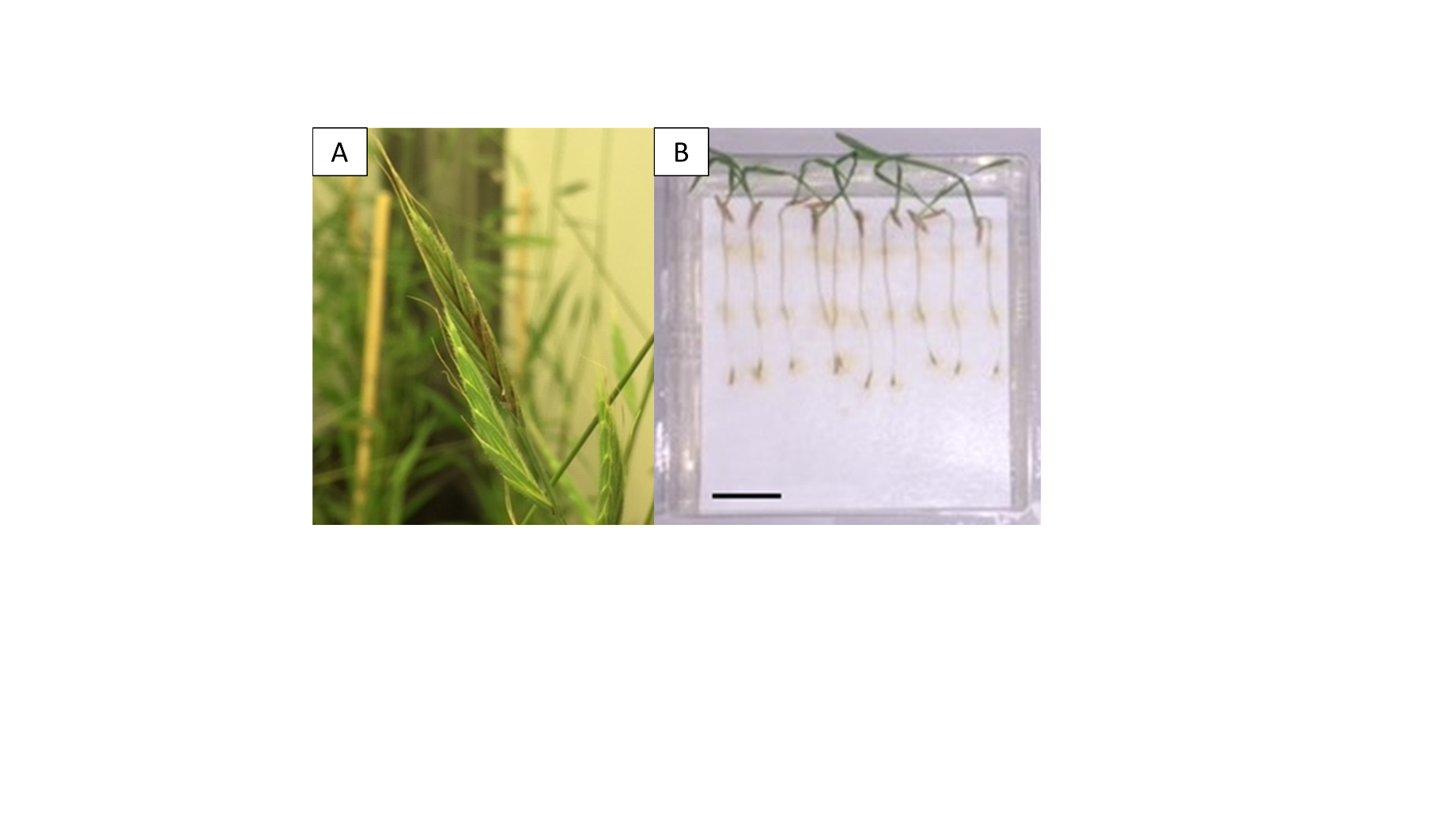


Supplementary Figure S1: *F. graminearum*-infected Bd3-1 florets and roots used in the RNA-seq assay. (**A**) Infected Bd3-1 spikelet at 5 dpi showing brown lesions on florets. (**B**) Bd3-1 seedling roots infected at 1 dpi showing three reddish/brown inoculated points from the *F. graminearum* slurry on each root. Roots were cut just below the seed for each plant. Scale bar = 2 cm. The scale bars was made with ImageJ: (Abràmoff et al., 2004)

The data described and presented in this study are also found at (Haidoulis and Nicholson, 2022a) and (Haidoulis and Nicholson, 2022b).

References

ABRÀMOFF, M. D., MAGALHÃES, P. J. & RAM, S. J. 2004. Image processing with ImageJ. *Biophotonics international,* 11**,** 36-42.

HAIDOULIS, J. F. & NICHOLSON, P. 2022a. Tissue-Specific Transcriptome Responses to Fusarium Head Blight and Fusarium Root Rot. *bioRxiv*.

HAIDOULIS, J. F. & NICHOLSON, P. 2022b. The Transcriptome Responses of Fusarium Head Blight and Fusarium Root Rot in B. distachyon. Dryad. doi.org/10.5061/dryad.cz8w9gj6k
